# Errata

**Published:** 1783

**Authors:** 


					f ? w>
/, i, for Andrew read Andrea.
END of VOL. IV.

				

